# Severe mpox in patients with advanced AIDS: long-term disease and fatal outcome

**DOI:** 10.1590/0037-8682-0228-2023

**Published:** 2023-11-10

**Authors:** Luís Arthur Brasil Gadelha Farias, Pablo Eliack Linhares de Holanda, Ana Danielle Tavares da Silva, Karene Ferreira Cavalcante, Marina Catunda Pinheiro Jucá, Lauro Vieira Perdigão, Lisandra Serra Damasceno

**Affiliations:** 1 Hospital São José de Doenças Infecciosas, Fortaleza, Ceará, Brasil.; 2 Programa de Residência Médica em Doenças Infecciosas, Escola de Saúde Pública do Ceará, Fortaleza, Ceará, Brasil.; 3 Programa de Pós-graduação em Saúde Pública, Faculdade de Medicina, Universidade Federal do Ceará, Fortaleza, Ceará, Brasil.; 4 Laboratório Central de Saúde Pública do Ceará, Fortaleza, Ceará, Brasil.; 5 Universidade de São Paulo, São Paulo, Brasil.

**Keywords:** Mpox, HIV, Immunosuppression

## Abstract

Herein, we report two cases of severe mpox in patients with advanced acquired immunodeficiency syndrome from Brazil who developed atypical lesions and prolonged illness, one of whom had a fatal outcome. Both patients experienced serious complications involving the perianal and genital regions and prolonged disease with persistent viremia.

## INTRODUCTION

The mpox virus is a microorganism belonging to the genus *Orthopoxvirus*, family *Poxviridae*, composed of enveloped double-helix DNA. The virus was first identified in 1957 during an investigation of dermatological lesions in monkeys infected in Denmark^1^. In 1970, the first human case was reported in the Democratic Republic of the Congo^2^. Similar to smallpox, mpox causes a significantly lower mortality^1^ ^,^ ^2^.

Mpox is an emerging human disease in countries outside Africa. In April 2022, autochthonous cases outside Africa began emerging. In May 2022, the World Health Organization (WHO) raised an alert regarding human mpox transmission, and in July, the disease was declared a global health emergency^2^ ^,^ ^3^. The first case of mpox in Latin America was confirmed on May 18, 2022, in Mexico^4^. In Brazil, the first case of mpox occurred in São Paulo in a 41-year-old patient who had traveled to Portugal and Spain^5^. As of February 17, 2023, more than 50,000 cases have been reported, with 73 deaths^3^ ^,^ ^6^. Brazil ranked second in the number of cases, with 10,825 confirmed cases and 15 deaths^6^.

This article reports two cases of severe human mpox in patients with advanced acquired immunodeficiency syndrome (AIDS) who were hospitalized at the Hospital São José de Doenças Infecciosas. The hospital is a reference healthcare unit for infectious diseases. This study was part of a cohort study approved by the Research Ethics Committee of the Hospital São José de Doenças Infecciosas (Protocol No: 5.857.478) and conducted in accordance with the 1964 Declaration of Helsinki and its later amendments. 

## CASE 1

**FIGURE 1:**
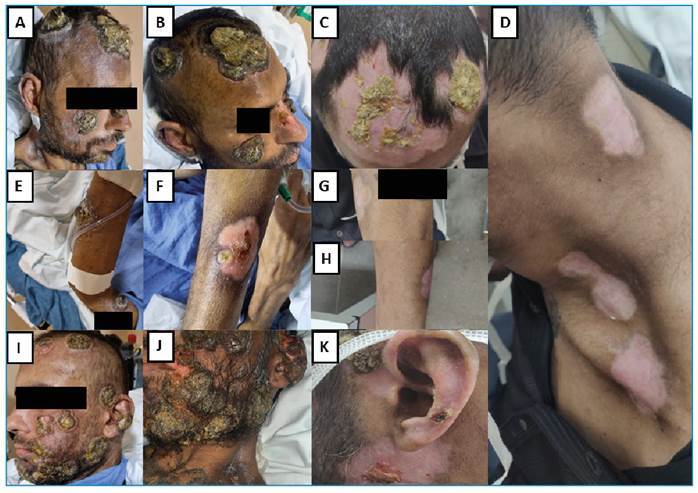
Ulcer lesions with a necrotic base and exophytic central crust on the face, affecting the frontal, temporal, infraorbital, parotideomasseteric, and left auricular regions **(A and I)** and left upper limb **(E)**. During treatment with tecovirimat, a reduction in the diameter of the crusts in the frontal and temporal regions **(B)** and healing area with reduction of the crust were noted. Appearance of a new lesion with a necrotic center in the left upper limb **(F)**. After approximately 45 days of tecovirimat treatment, the appearance of healing areas with a reduction in the size of the crusts on the face and scalp (C), complete healing of the lesions on the left upper limb and parotideomasseteric and left auricular region **(D, G, H, K)**.

A 39-year-old man with AIDS presented to the emergency room (ER) with painful perianal-umbilical vesicular lesions associated with purulent discharge in September 2022. The patient was a man who had sex with men (MSM) and had been infected with human immunodeficiency virus (HIV) for 13 years but had not adhered to antiretroviral therapy (ART). The CD4^+^ cell count was 20 cells/mL, and the HIV load was 1,019 copies/mL. After 3 weeks, new bullous and papulo-crusted lesions appeared on the limbs and face. He was hospitalized with ulcers and cornu cutaneum-like crusts on the face (Figure 1A, E, I ), and purulent discharge persisted in the anal canal. Real-time reverse transcription polymerase chain reaction (RT-qPCR) was performed for the mpox virus from the vesicular lesions on the limbs and face. The results were positive. Antibiotic therapy was initiated with ceftriaxone and oxacillin as well as ART (tenofovir plus lamivudine 300 mg and dolutegravir 50 mg per day). However, owing to the worsening of the cutaneous and mucosal lesions, antibiotic therapy was scaled up to vancomycin and piperacillin-tazobactam. Pelvic computed tomography (CT) showed a laminar subcutaneous collection in the posterior pelvic wall measuring 9.9 x 2.2 cm. After 21 days, clinical evaluation showed improvement of the anal abscess. However, the skin lesions and new disseminated lesions persisted (Figure 1B, F, J). Five months later, owing to the emergence of new lesions and the lack of significant improvement in the previous lesions, an oral tecovirimat regimen (1,200 mg/day) was administered for 2 weeks. This drug was only available in Brazil during this period. Six months after mpox onset, the CD4^+^ cell count was 64 cells/mL, and the viral load was undetectable. The skin lesions were crusted on the upper limbs and scalp. RT-qPCR for the mpox virus was repeated until the sixth month when new skin lesions surged and persisted with low cycle threshold values. Finally, the lesions began to heal (Figure 1C, D, G, H, K), and the patient was discharged in March 2023.

## CASE 2

**FIGURE 2:**
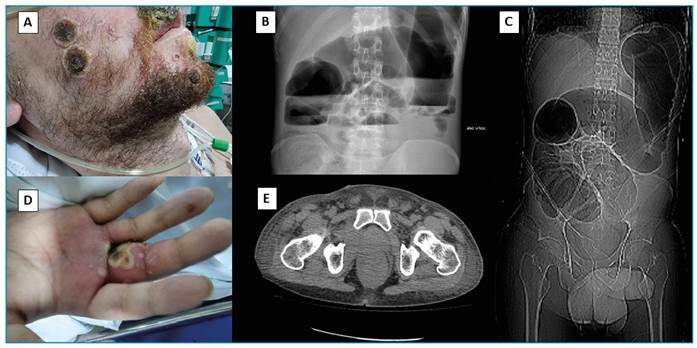
Ulcerated lesion with central necrosis and purulent border on the right cheek **(A)** and proximal phalanx on the upper lip of the third finger on the right hand (D). Abdominal radiography in orthostasis shows gaseous distension of the intestinal loops with Peer Review air-fluid level **(B)**, and abdomen computed tomography shows distension of the colon, with heterogeneous content forming a level and irregular parietal thickening with reduced lumen transition between the sigmoid and the rectum **(C and E)**.

In October 2022, a 29-year-old man, MSM, with AIDS, presented to the ER with polymorphic bullous skin lesions on the upper limbs and dorsum (Figure 2A, D). In addition, he had a violaceous lesion on the anterior surface of the left foot, suggestive of Kaposi’s sarcoma. He had been infected with HIV for 9 years and had not adhered to ART. The patient had a CD4^+^ cell count of 61 cells/mL and an HIV load of 41 copies/mL. RT-qPCR was performed to detect the mpox virus in a sample of vesicular lesions on the upper limbs, and the result was positive. Histopathological examination of the violaceous lesion confirmed the presence of Kaposi’s sarcoma. Therefore, analgesic treatment was initiated. One month later, the patient was hospitalized because of coalescent, painful, and disseminated lesions on the upper lip, back, upper and lower limbs, and the genital region. Abdominal CT revealed marked distension of the colon, irregular and heterogeneous parietal thickening between the sigmoid and rectum, luminal reduction and densification of the local adipose planes, and enlargement of numerous lymph nodes in the mesorectal space (Figure 2B, C, E). The patient underwent laparotomy. A hardened endoluminal lesion in the rectosigmoid was visualized, and a colostomy was performed. Five days after the surgery, the patient developed sepsis. He received vancomycin and meropenem. *Enterococcus faecium* vancomycin-resistant bacteria were isolated from blood cultures. Despite receiving antimicrobial therapy, the patient died of refractory septic shock on day 10 after surgery. 

## DISCUSSION

Studies have demonstrated that the clinical and epidemiological features of the new mpox differ from those of the endemic mpox in Africa. Usually, the infection is self-limiting, and death is rare^1^ ^-^ ^7^. Therefore, the disease is characterized as a zoonotic infection, and person-to-person transmission has rarely been described. 

According to the WHO, deaths have been reported in all six continents of the world^2^ ^,^ ^8^. In Brazil, the first reported death occurred in a 41-year-old immunocompromised patient with diffuse large B-cell lymphoma with metastases to the spine, skull, and liver^9^. 

Hospitalization is usually associated with secondary bacterial infections, intense pain, and edema, mainly in the perineal and genital regions. A series of six hospitalized patients from Berlin presented with intense anal pain due to proctitis and anal and rectal ulcers. Two patients had an HIV infection^10^. In Brazil, 109 individuals were coinfected with HIV in a cohort of 208 patients with confirmed mpox. In this study, the median CD4^+^ cell count was > 500 cells/mL, and most patients were virologically suppressed. A total of 19 patients were hospitalized because of bacterial superinfections, paraphimosis, and pain. No deaths were observed^8^.

Govind et al. recently reported three cases of severe mpox in patients with AIDS, causing debilitating lesions, complications, and death despite initiating ART^11^. In the USA, 57 patients were hospitalized with severe manifestations of mpox, 47 (82.5%) had HIV infection, and 31 had CD4^+^ cell counts of < 50 cells/mL. Five deaths occurred because of mpox^12^. 

Mitjà et al. published the largest mpox series with 382 patients with HIV. Severe mpox is characterized by widespread, large necrotizing, and coalescing skin lesions. The median duration of skin lesions in individuals with 100 or more lesions was 40 days. Additionally, 27 patients died, with a median CD4+ cell count of 35 and a high HIV load. In the context of advanced AIDS, mortality due to mpox can affect up to 15% of the patients^7^.

Herein, we describe two severe cases of mpox in patients with advanced AIDS. Both patients experienced serious complications, such as prolonged disease with crusted lesions (> 150 days in Case 1) and persistent viremia of mpox. The other patient died of intra-abdominal bacterial sepsis. Surgery for intestinal obstruction can lead to bacterial translocation and sepsis. Generally, the complete resolution of lesions occurs within 50 days from the onset of symptoms^7^. The rash duration ranged between 50-100 days, and deaths were observed in individuals with a CD4+ cell count < 200 cells/mm3 and in some individuals with a high viral load^7^. Thus, immunosuppression may corroborate the long-term atypical manifestations and more severe forms of the disease^7^ ^-^ ^9^.

Unfortunately, the mpox vaccine was unavailable in Brazil in both cases. The mpox vaccine (MVA-BN Jynneos®) was acquired by the Ministry of Health in March 2023^13^. Globally, only a few patients have access to specific therapies for mpox disease. Drugs such as cidofovir and tecovirimat have been used in the USA and Europe to treat severe cases of mpox; however, these drugs have not yet been tested in phase III clinical trials^14^. In Brazil, the National Health Surveillance Agency approved tecovirimat for compassionate use in August 2022. The criteria for using tecovirimat included severe and disabling disease and risk of death when there was no other therapeutic alternative. 

In this report, only Case 1 received tecovirimat treatment. We cannot confirm that the therapeutic response to tecovirimat was effective and the main factor involved in the clinical improvement in Case 1. The expectation is that using tecovirimat for 5 days will be sufficient to induce a clinical response. However, maintaining the levels of it for 2 weeks allows for better development of humoral immunity and longer viral elimination^12^. Further studies are required to establish the efficacy of this drug against mpox severity and mortality^14^ **.** ART and immune recovery may also contribute to clinical improvements. 

## INFORMED CONSENT

This study was part of a cohort study approved by the Research Ethics Committee of the Hospital São José de Doenças Infecciosas and was conducted in accordance with the ethical standards set forth in the Declaration of Helsinki 1964 and its later amendments or equivalent ethical standards (IRB no. B-2301-805-701). The patients signed a consent form for this report.
